# The Effect of Statins on Carotid Intima–Media Thickness and C–Reactive Protein in Type 2 Diabetes Mellitus: A Meta–Analysis

**DOI:** 10.3390/jcdd11090276

**Published:** 2024-09-04

**Authors:** Given Reneilwe Mashaba, Wendy Nokhwezi Phoswa, Kabelo Mokgalaboni

**Affiliations:** 1 Department of Life and Consumer Science, College of Agriculture and Environmental Sciences, University of South Africa, Florida Campus, Roodepoort 1710, South Africa; phoswwn@unisa.ac.za; 2 DIMAMO Population Health Research Centre, University of Limpopo, Sovenga, Private Bag X1106, Polokwane 0727, South Africa

**Keywords:** type 2 diabetes mellitus, carotid intima–media thickness, statins, C-reactive protein

## Abstract

Background. The effect of statins on CIMT progression and C-reactive protein (CRP) in T2DM patients is widely reported. However, some studies demonstrated no effect of statins on CIMT and CRP in T2DM patients, while others reported otherwise. Thus, the current study comprehensively and quantitatively analyzes data from previous studies to evaluate the overall effect of statins on CIMT and CRP in T2DM to rule out any inconsistencies observed in previous clinical evidence. Therefore, the aim of this meta-oanalysis was to evaluate the effect of statins on CIMT progression and CRP in T2DM. Methods. A comprehensive search for studies was performed using PubMed, Scopus, Web of Sciences, and the Cochrane Library, for publications from their inception to 16 July 2024. The meta-analysis was conducted using Jamovi (version 4.2.8) and Review Manager (version 5.4), with the overall effect sizes reported as standardized mean differences (SMD) and 95% confidence intervals (CI). Results. Evidence from eleven studies (fifteen statin dosages) that met the inclusion criteria with a sample size of 983 T2DM patients on statin treatment was analyzed. The overall effect size from the random effect model meta-analysis showed a reduction in the CIMT status amongst T2DM patients post-statin treatment compared to at baseline [SMD = −0.47, 95%CI (−0.76, −0.18), *p* = 0.001]. Furthermore, there was a reduction in the level of CRP in T2DM patients post-treatment [SMD = −1.80, 95% CI (−2.76, −0.84), *p* < 0.001]. Conclusions. Evidence gathered in this study suggests that statin therapy effectively reduces CIMT and CRP levels among patients living with T2DM. Interestingly, this evidence suggests that 20 mg of atorvastatin is more effective in reducing CIMT and CRP. Therefore, we recommend conducting further trials with larger sample sizes and proper methodology for T2DM.

## 1. Introduction

Cardiovascular diseases (CVDs) are the main cause of mortality in diabetes mellitus (DM) patients [1,2]. The prevalence of CVD amongst DM patients is estimated to be around 32.2% [3]. CVDs are characterized by endothelial dysfunction (ED), which promotes atherosclerosis [4]. Endothelial dysfunction associated with DM is a reversible condition that precedes atherogenic plaque formation and atherosclerosis [5,6]. The severity and progression of carotid endothelial atherosclerosis can be assessed by a non-invasive ultrasonography measurement of the carotid intima–media thickness (CIMT) [7]. CIMT correlates positively with the prevalence of CVDs and risk factors such as dyslipidemia, making it valuable in predicting CVD; hence, it is increasingly used as an intermediate endpoint in clinical trials [8,9].

The relationship between type 2 diabetes mellitus (T2DM) and ED is more complex and involves mechanisms relating to hyperglycemia and insulin resistance, which are common features of DM [10]. Hyperglycemia and insulin resistance reduce the production and availability of nitric oxide (NO) [11], thus inducing ED [12,13]. The existing research has reported the benefits of statins, lipid-lowering agents, in improving ED among T2DM patients [14,15]. While the effects of statin in T2DM, especially on lipid profiles, are acknowledged, the overall impact on C-reactive protein (CRP) in T2DM is still not clear due to inconsistencies arising from various study designs [16]. For instance, some trials revealed that atorvastatin does not reduce CRP levels in T2DM patients [17,18,19,20], while other studies do not confirm whether the baseline CRP concentration influences the vascular benefits of statin therapy [21,22].

In contrast, a meta-analysis revealed that statins could reduce inflammatory markers, including interleukin-6 and CRP [21]. However, it is essential to note that evidence was collected from a few trials, which might limit the interpretation of such evidence. In other studies, statins were reported to reverse EDs in non-diabetic participants through the upregulation of endothelial NO synthesis expression and the inhibition of superoxide synthesis [23,24]. However, the clinical evidence for T2DM has demonstrated null findings for NO levels [25,26]. It is also unclear how statin impacts CIMT progression in T2DM patients, as contradictory results have been reported. Additionally, the effect of statins on CIMT progression in T2DM patients remains inconclusive as some studies reported no effect and others suggest otherwise [27,28]. This uncertainty is further compounded by the limited number of randomized control trials (RCTs) available on this topic, many of which were conducted with small sample sizes, which might be underpowered.

While the mechanism of statin in the modulation of endothelial function is not clear, it is important to note that statins as lipid-lowering agents inhibit the hydroxymethylglutaryl Coenzyme A reductase (HMG-CoA reductase) and enzyme that promotes the conversion of mevalonate into cholesterol [29]. If not inhibited, HMG-CoA reductase increases cholesterol synthesis, which can exacerbate insulin resistance. Therefore, statins may improve insulin sensitivity by blocking HMG-CoA reductase and lowering cholesterol [30].

Considering the inconsistencies about the impact of statins in T2DM on CIMT and CRP, it is important to evaluate their overall effect using high-quality evidence. Therefore, the current study aims to comprehensively and quantitatively analyze available data to evaluate the effect of statins on CIMT progression and CRP in T2DM and to rule out any inconsistencies observed in previous clinical evidence.

## 2. Methods

This study adhered to Cochrane guidelines [31] and is reported according to updated Preferred Reporting Items for Systematic Reviews and Meta-Analyses (PRISMA) guidelines [32]. The study is reported according to the PRISMA checklist in Supplementary File S1. The protocol for this meta-analysis was registered at PROSPERO, registration number (CRD42024581929).

### 2.1. Ethics Approval

The present study synthesized peer-reviewed published data from scientific journals and thus did not require ethical approval from an institutional ethics committee. However, the analyzed studies received ethical approval and patient consent from their institutional ethical committees, thus ensuring ethical standards for the present study. 

### 2.2. Eligibility Criteria

#### 2.2.1. Inclusion Criteria

All studies that met our population, intervention, control, and outcomes (PICO) criteria were included. The PICO were defined as follows: baseline data of patients with type 2 diabetes (P), any form of statin as intervention (I), post-treatment data (C), and changes in CIMT and CRP (O). In cases where a study was conducted using two different dosages of statin, both arms were considered as different studies. No restriction was applied in terms of language in the search, and any study published in a language other than English was translated using an online translator.

#### 2.2.2. Exclusion Criteria

Studies that did not measure CIMT and CRP, abstracts, letters, reviews, conference proceedings, and studies without enough data were all excluded. Studies using animal models of T2DM and evidence from grey literature were all deemed irrelevant and thus excluded.

### 2.3. Information Source and Search Strategy

A comprehensive literature search was performed using PubMed, Scopus, Web of Sciences, and Cochrane Library databases for studies published from their inception to 16 July 2024. The following medical subject heading (MeSH) terms were used to identify relevant studies: “statins” and associated examples such as “atorvastatin”, “pitavastatin”, “pravastatin”, “rosuvastatin”, “simvastatin”, “lovastatin”, “fluvastatin”, “cerivastatin”, “diabetes mellitus”, “type 2 diabetes mellitus”, “carotid intima media-thickness”, “CIMT”, “C-reactive protein”, and “CRP”, which were combined using OR and AND as Boolean operators. Reference lists of retrieved studies were also screened for additional relevant studies. Two researchers (K.M. and G.R.M.) independently screened titles, abstracts, and keywords of all retrieved studies, and discrepancies were resolved through discussion and re-evaluation of the study.

### 2.4. Data Items and Extraction

Data from studies that met the inclusion criteria were extracted by independent researchers (R.G.M. and K.M.). A senior researcher (K.M.) formulated a Microsoft 365 Excel (Version 2307) data extraction sheet, which was subsequently shared with the other researchers (R.G.M. and W.N.P.) before the extraction process. Upon agreement on the structure of the data extraction template, two researchers (R.G.M. and K.M.) independently extracted data from each study and recorded details such as author’s surname, publication year, country, study design, number of males and females and their percentage, sample size of the included T2DM patients, baseline mean age within the statin group, intervention and duration, baseline body mass index (BMI), type of statins and dosage used, and summary of main outcomes. The continuous data extracted for the main outcomes and anthropometric parameters were means ± standard deviation (SD) of CIMT and CRP at baseline and post-treatment. K.M. and R.G.M. independently conducted the data extraction to control potential biases in the extraction process. However, in cases of discrepancies, a third independent researcher, W.N.P., was invited to reassess the study or data in question.

### 2.5. Risk of Bias in Individual Studies

The risk of bias (ROB) across all studies that met the inclusion criteria was assessed according to the guidelines of the Cochrane risk of bias tool [33,34]. The following five domains were evaluated: bias arising from the randomization process, deviation from intended intervention, missing outcome data, bias in the measurement of the outcome, and selective outcome reporting. The evaluated domains were assigned as “Low”, “High”, “No information”, or “Uncertain” according to the criteria of the Cochrane guidelines. A study was classified as low risk if all domains were judged as having a low risk of bias and classified as being of some concern if one domain was judged as having some concern. Two independent researchers (K.M. and G.R.M.) made the overall judgment. In the event of disagreement, a third researcher (W.N.P.) judged the domain in question.

### 2.6. Data Synthesis and Statistical Analysis

The meta-analysis was conducted using Jamovi (version 4.2.8; Computer software, Sidney, Australia) and Review Manager (version 5.4; Cochrane Collaboration, Oxford, UK). Means and SD of the outcome measures (CIMT and CRP) reported for the statin treatment at baseline and post-treatment were used to obtain the overall effect size. In cases where the study reported median and interquartile range (IQR), an online calculator was used to estimate mean and SD, https://www.math.hkbu.edu.hk/~tongt/papers/median2mean.html (accessed 18 July 2024). Alternatively, SD was estimated using the standard error of the mean (SER) = SD/√n when the trial reported an SER. In cases where the treatment group was reported in different statin dosages, the mean and SD of the groups were combined using CombineMeanSD (statstodo.com) (accessed 18 April 2024). The overall effect sizes for all effect measures were reported as standardized mean differences (SMDs) and 95% confidence intervals (CIs). Random effect models were used due to expected moderate heterogeneity. We further assessed heterogeneity between studies through the *I*^2^ statistics and the Q test. An (*I*^2^ > 50%) was considered moderate heterogeneity [35,36]. Publication bias was assessed by visualizing the funnel plot and using Beggs and Mazumdar rank correlation and Egger’s regression test. A *p*-value greater than 0.05 for these tests implied no evidence of publication bias. A fail-safe N test was used to assess the robustness of our meta-analysis to publication bias. A large fail-safe N indicated that the meta-analysis results were robust to publication bias, while a small fail-safe N raised concerns about the reliability of the findings. Moreover, subgroup analysis was performed to evaluate the source of heterogeneity. This was based on forms of statin, dosage, age, and continent of publication.

## 3. Results

### 3.1. Literature and Sources

**Figure 1:**
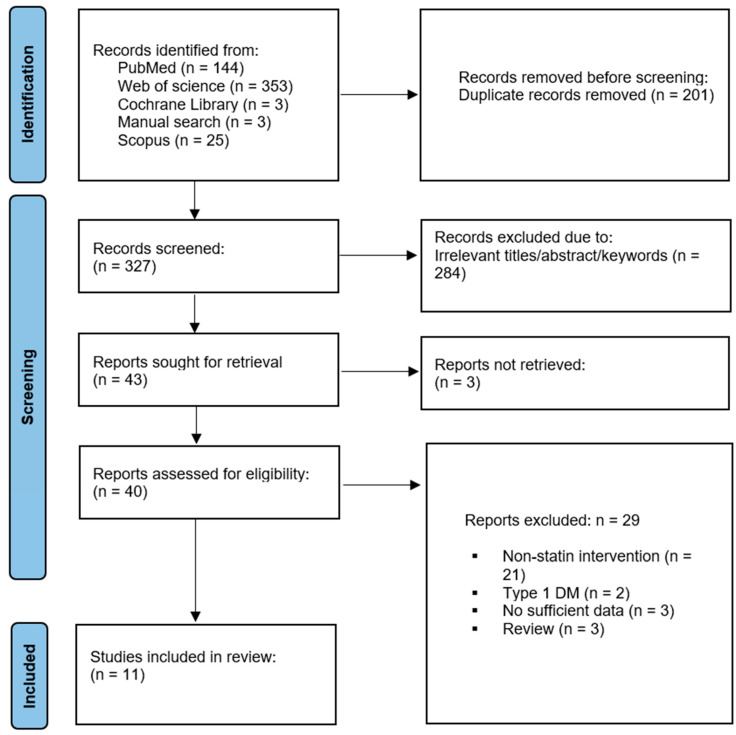
Flow diagram for the studies evaluated and included in systematic review and meta-analysis.

At least 525 studies were identified from the four databases and a manual bibliography search yielded three relevant studies (Supplementary File S2, Table S1). Of these records, 201 were duplicates, and 284 had irrelevant titles, abstracts, and keywords and thus were excluded. Three articles’ full text could not be obtained, and requests via emails were made to obtain these files; however, no response was received from the corresponding authors. Hence, they were not evaluated in full text. Therefore, 40 records were retrieved and assessed for eligibility, and 29 were excluded for the following reasons: T1DM, no statin treatment, reviews, and insufficient data. Eleven studies with fifteen treatment arms [20,37,38,39,40,41,42,43,44,45,46] were deemed relevant (Figure 1).

### 3.2. Basic Characteristics of Included Studies

Table 1 presents the general characteristics of the included studies. The present review and meta-analysis included eleven studies with fifteen treatment arms (Figure 1) that met the inclusion criteria. These studies comprised 983 T2DM patients on statin treatment [20,37,38,39,40,41,42,43,44,45,46]. Of these studies, ten were randomized controlled trials, and one was a prospective observational study. The included studies were conducted in the Netherlands [40], the United States of America [38,39], Greece [43], China [37,44,45], India [41], Japan [42,46], and Turkey [20]. Although not all studies reported gender distribution, about 444 (47%) of the sample patients were males and 509 (53%) were females. The mean age of included T2DM patients on statin was 59.86 years. While one study [44] did not report the exact body mass index (BMI), the BMI from ten studies [20,37,38,39,40,41,42,43,45,46] was 24.48 ± 2.32 kg/m^2^. All studies reported the effect of statins on the CIMT, while seven studies included reports on the effect of statins on CRP. The duration of statin treatment ranged between two and a half and thirty-six months across the included studies. The types of statin treatments that were used included simvastatin [40,45,46], atorvastatin [20,37,38,43,44,45], pitavastatin [42], rosuvastatin [41], and pravastatin [42,46]. However, one trial [39] did not specify the form of statin treatment used. The dosage of statins ranged from as low as 10 mg to 80 mg per day.

### 3.3. Risk of Bias (ROB) Assessment

The evidence gathered in this study showed some concerns in different domains. For instance, there was no information about the process of randomization (D1) in at least eight studies [37,38,39,41,43,44,45,46] with ten treatment arms. Only two studies [40,42] with three statin doses reported methods of randomization, such as computer-generated randomization through a block method. However, one study was not a randomized trial [20]. Overall, two studies with three statin doses [40,42] were classified as good quality as all domains were classified as low risk. One study was classified as high risk as it was not a randomized trial [20], while eight studies with eleven statin treatments were classified as fair due to some concerns in other domains, mainly the process of randomization (Supplementary File S2, Figures S1 and S2).

### 3.4. Effect of Statins Treatment on CIMT in T2DM Patients

**Figure 2:**
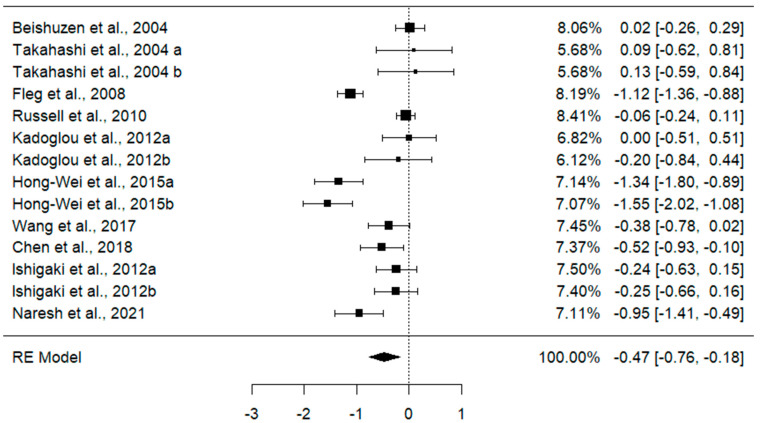
Effect of statins on carotid intima–media thickness in T2DM patients at baseline versus post-treatment [37,38,39,40,41,42,43,44,45,46]. Data are reported as standardized mean difference and 95% confidence intervals, *p* ˂ 0.001.

Ten studies [37,38,39,40,41,42,43,44,45,46] with fourteen different statin doses and a sample size of 909 patients living with T2DM were examined to assess the impact of statins on carotid status. The overall effect size from the random effect model meta-analysis showed a reduction in the level of CIMT in T2DM patients post-treatment compared to at baseline [SMD = −0.47, 95%CI (−0.76, −0.18), *p* = 0.001] (Figure 2). The magnitude of the effect of statin was moderate (Cohen’s d = 0.5). Of concern was an elevated level of heterogeneity among the included studies (*I*^2^ = 87.83%, *p* ˂ 0.001) and Q =109.83 (Figure 2). 

### 3.5. The Effect of Statin on C-Reactive Protein in T2DM

**Figure 3:**
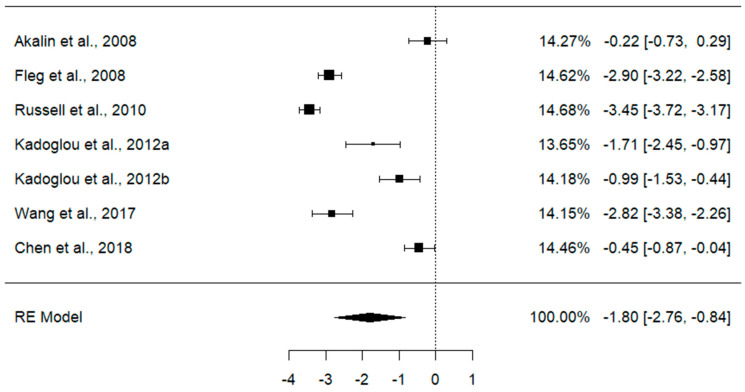
Effect of statins on C-reactive protein in T2DM patients at baseline versus post-treatment [20,37,38,39,43,44]. Data are presented as standardized mean differences and 95% confidence intervals, *p* ˂ 0.001.

The effect of statins on CRP was analyzed from six studies [20,37,38,39,43,44] with a sample size of 579 T2DM patients on seven different statin doses. The overall effect size from the random effect model meta-analysis showed a reduction in the level of CRP in T2DM patients post-treatment compared to at baseline [SMD = −1.80, 95% CI (−2.76, −0.84), *p* ˂ 0.001] (Figure 3). Interestingly, the magnitude of this effect of statins on CRP was significantly high (Cohen’s d > 1). However, these studies showed a high level of heterogeneity (*I*^2^ = 97.15%, *p* ˂ 0.0001), and Q = 248.74.

### 3.6. Subgroup Analysis

Subgroup analysis on CIMT based on the form of statin revealed that atorvastatin has a significant effect in reducing CIMT in T2DM [SMD = −0.41, 95%CI (−0.80, −0.03), *p* = 0.04)]. Rosuvastatin also significantly reduced CIMT [SMD = −0.95, 95%CI (−1.41, −0.48), *p* ˂ 0.0001]. No significant effect was observed on simvastatin [*p* = 0.38], rosuvastatin [*p* = 0.38], or pitavastatin [*p* = 0.22] (Supplementary Figure S2). Additionally, 20 mg of atorvastatin as treatment was deemed effective in reducing CIMT [SMD = −0.74, 95%CI (−1.31, −0.17), *p* = 0.01]. A dosage of 20 mg/day of rosuvastatin showed a significant effect; this was evidenced by one trial. The 2, 10, and 80 mg doses showed no significant effect (Supplementary Figure S3). A subgroup analysis on the continent of publication was conducted to find out if the place of publication introduced biases, and we found that studies published in Asia reported a significant effect of statins on CIMT [SMD = −0.59, 95%CI (−0.95, −0.22), *p* = 0.002] compared to Europe [*p* = 0.73] and America [*p* = 0.27] (Supplementary Figure S4). Lastly, the age of T2DM patients was also used to perform subgroup analysis, and we found that patients above the age of 60 had significantly decreased CIMT [SMD = −0.56, 95%CI (−0.94, −0.17), *p* = 0.005] compared to those below the age of 60 [SMD = −0.49, 95%CI (−0.57, −0.08), *p* = 0.008] (Supplementary Figure S5). Regarding CRP, a subgroup analysis based on the forms of statin showed a significant effect when atorvastatin was used [SMD = −1.61, 95%CI (−2.89, −0.33), *p* = 0.01] (Supplementary Figure S6). Regarding the dosage of statin, only 80 mg reduced CRP [*p* ˂ 0.00001] (Supplementary Figure S7). However, for CRP based on the age of patients, it was shown that those below the age of 60 had a significant decrease in CRP [*p* = 0.0002] compared to those above the age of 60 [*p* = 0.004] (Supplementary Figure S8). Based on the continent of publication, only evidence from Europe showed a significant reduction in CRP [SMD = −1.3, 95%CI (−2.0, −0.60), *p* = 0.0003] (Supplementary Figure S9).

### 3.7. Publication Bias

**Figure 4:**
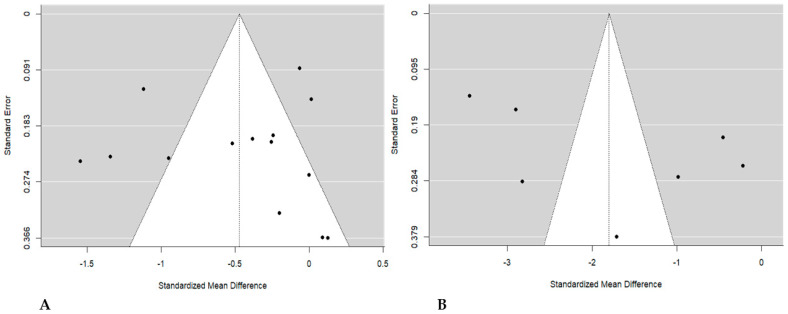
Funnel plots showing assessment of publication bias. (**A**) Publication bias for carotid intima–media thickness; (**B**) publication bias for trials evaluating C-reactive protein.

Assessment of publication bias through visual inspection of funnel plots for studies that evaluated CIMT indicated no asymmetrical shape, suggesting no bias (Figure 4A). This was confirmed further by statistical tests from the Beggs and Mazumdar rank correlation (*p* = 0.830), and the Egger regression test (*p* = 0.566) indicated no publication bias. Interestingly, the fail-safe N test also supports the above results (N = 381.000, *p* ˂ 0.001), suggesting that the obtained results are robust to publication bias. Similarly, the funnel plot for the studies that evaluated CRP revealed no bias (Figure 4B). This was confirmed by Begg and Mazumdar rank correlation and Egger’s regression tests (*p* = 0.773 and *p* = 0.351, respectively). The fail-safe N test supports the above findings (N = 1472.000 and *p* < 0.001).

## 4. Discussion

Cardiovascular disorders and mortality arising from T2DM are increasing globally, with the highest burden observed in low-income countries [1,2]. Different pharmacological regimens have been used over the years to control chronic hyperglycemia; however, there remains a risk of chronic inflammation and cardiovascular events in T2DM [47]. Therefore, there is an urgent need to contain these rising rates of CVDs and associated mortality. This systematic review and meta-analysis investigated the effect of statin treatment to curb the associated CVD complications amongst patients living with T2DM on the CIMT and selected marker of inflammation, CRP. The results obtained in this study revealed a significant reduction in the CIMT in T2DM patients following statin treatment compared to those at baseline with a magnitude of effect size of 0.5. They suggest a moderate effect size.

Additionally, the statin treatment substantially decreased the concentration of CRP, a central marker of inflammation in T2DM with a large effect size; Cohen’s d > 1. CIMT is regarded as a sensitive biomarker of atherosclerosis and can predict future clinical cardiovascular events. While the current findings suggest that statins can reduce CIMT in T2DM, it is important to note that other clinical trials dispute these findings as they reported contradicting findings, where CIMT was not significantly different between baseline and post-statin exposure in T2DM patients. For instance, Ishigaki et al. in 2014 reported that pravastatin at 10 mg/day revealed no significant difference in CIMT between baseline and post-treatment [42]. However, the same trial showed that 2 mg of pravastatin could reduce CIMT in T2DM. Another trial that used simvastatin at 20 mg also reported no effect on CIMT in T2DM [40]. The latter findings contradict our current results. Kadoglou et al. in 2012 also reported contrasting results, as a low dose of atorvastatin yielded no effect; however, a higher dose showed a beneficial effect on CIMT [43]. These results suggest that the effect of statin on CIMT might be dose-dependent.

Although not in diabetes, other studies have reportedly shown that statins significantly reduce CIMT [48,49]. Contrasting findings have been reported by various trials, which showed no effect of statin on CIMT in T2DM [20,40,42,43]. Despite these conflicting findings from other trials, our results are supported by previous meta-analyses in T2DM, which reported a reduction in CIMT following atorvastatin treatment [27]. However, it is important to note that this study was conducted in T2DM patients of Chinese origin, which might limit the translatability of the results to the global T2DM population. Interestingly the same trend is observed in T2DM patients on different forms of statins [37,38,39,41,44,45,46,50].

Previous evidence suggests that intensive lipid-lowering drugs can reduce the rate of change in CIMT; however, this study focused on statins and other lipid-lowering drugs in patients other than T2DM patients [51]. Similarly, a review by Mookadam et al. reported a positive effect of statins on the advancement of atherosclerosis with high doses, resulting in the reversal of carotid atherosclerotic disease, and lower-dose treatments only slowed down its progression. However, the study was not conducted in the T2DM population [52]. Given that the rate of change of CIMT reflects a change in CVD events [51] and the risk of atherosclerotic CVD events that T2DM patients have [3,53], treatments aimed at lowering this risk, such as statins, irrespective of effect size, should be taken into consideration to minimize the associated risk. While the exact mechanism by which statin lowers CIMT is not well documented, previous evidence reports that this might be mediated by its lipid-lowering properties [54]. Briefly, statin’s lipid-lowering mechanism is achieved by inhibiting HMG-CoA reductase, a key precursor compound in cholesterol biosynthesis [55]. This results in reduced cholesterol synthesis and a decrease in LDL levels, which subsequently reduces the development of atherosclerotic plaque in the carotid arteries [55]. CIMT progression has been reported to be more rapid in those with an inflammatory conditions, including T2DM patients [56]. CRP is one of the markers used to measure inflammation [57]. 

High CRP levels have been associated with CIMT progression. A study by Pirro et al. reported that high CRP levels (>3 mg/L) were associated with a 2.7-fold increased risk of having high CIMT (>1.25 mm); however, this was not in T2DM [58]. This suggests a direct proportional association, implying that a reduced CRP level would result in a reduced CIMT level. Any therapeutic measure that can reduce inflammation is deemed essential as it can alleviate further inflammation-induced complications. The meta-analysis revealed that statin treatment significantly reduces levels of CRP in T2DM, as revealed by an SMD of 1.96. According to Cohen’s criteria, the observed SMD effect was high, supporting the statin’s anti-inflammatory effect in T2DM [59]. A review by Kandelouei et al. reported similar findings in which statins decreased serum levels of CRP (SMD = 1.25) in patients with CVD (angina and heart failure); however, the study did not include T2DM [60]. The observed SMD in the present study compared to the study by Kandelouei suggests that statins may be more effective in T2DM than in other CVDs. Individuals on statin treatment are reported to have lower levels of inflammatory markers, including CRP, than those on a placebo [60,61]. Similarly, a study by Owens in 2012 reported that statins reduce CRP levels by 25–50% [62]. 

On the other hand, another study reported contradicting results as it revealed no effect of statin on CRP [63]. Similarly, Soedamah-Muthu et al. observed no effect following 10 mg of atorvastatin on CRP in T2DM [17]. In contrast, a study by Golia et al. in 2014 found that statins reduced levels of high-sensitivity CRP and other inflammatory biomarkers [64]. Although the present study did not analyze lipids, the anti-inflammatory property of statins seems to be attributed to their lipid-lowering properties [54]. In addition, statins have anti-inflammatory properties that inhibit the production of pro-inflammatory cytokines [65]. Moreover, the lipid-lowering effect of statins and anti-inflammatory properties may contribute to the regression or stabilization of CIMT [65].

### 4.1. Clinical Implications of the Study

For clinical set-ups (clinics and hospitals), the observed results suggest that statin treatment has beneficial effects in the management of cardiovascular-related complications in patients with T2DM. The observed effects of statin on CIMT and CRP further suggest that statin not only offers lipid-lowering properties but has additional effects in reducing inflammation and early atherosclerosis. Clinicians may consider the use of statin, especially atorvastatin (20 mg dosage), for T2DM patients at high risk for cardiovascular events. However, it is important for clinicians to consider patients’ basic characteristics, including age, gender, and potential statin intolerance, when prescribing these treatments. Moreover, health personnel should regularly monitor CIMT and CRP in T2DM patients on statin therapy. This could be valuable in evaluating the efficacy and adjusting therapeutic strategies accordingly. Applying the findings of the present study will not only add to the body of knowledge but could also reduce CVD-related mortality through the wider implementation of statin therapy, especially in low-income countries where the burden of CVD in T2DM is rapidly increasing. 

### 4.2. Strengths and Limitations of the Study

The present study analyzed evidence from twelve randomized controlled trials and one prospective observational study. However, it is important to note that the quality of studies was only regarded as good in two trials with three treatment arms. Individual assessment of risk of bias revealed a low risk of bias across all four domains except challenges with the randomization process, as noted in different studies. There was no risk of publication bias across those studies that assessed CIMT and CRP, indicating that the quality of the studies was satisfactory. Interestingly, two main databases were used by independent researchers to search for studies. 

We acknowledge some of the limitations within our research, notably, the scarcity of relevant trials, resulting in a small sample size comprising only 983 patients with T2DM on statin treatment. Additionally, the analyzed studies used diverse quantitative approaches, different types of statins, and differing treatment durations and dosages, potentially leading to statistical variations. Hence, the observed methodological variation led to statistical heterogeneity. The use of different forms of statins, dosages, and duration of intervention limited our interpretation of the effective dosage of statin. However, a detailed subgroup analysis was used to find the source of observed heterogeneity by exploring the effect of age, types of statins, dosages, and continent of publication on the overall effect size. The overall interpretation of the findings may be limited by the fact that two studies did not report the gender distribution of participants, and one did not report BMI. While meta-analyses of RCTs and observational designs are not generally performed, these can be carried out in certain cases [66].

## 5. Conclusions

Data reported in the present review and meta-analysis revealed a significant reduction in CIMT and CRP following statin treatment. This suggests that statin therapy effectively reduces the risk of CIMT-associated CVD and ameliorates inflammation in T2DM. Interestingly, based on our findings, 20 mg of atorvastatin was more effective in reducing CIMT and CRP among T2DM patients. Administering statin should be accompanied by regular assessment of CIMT and CRP levels to monitor the inflammatory and atherosclerotic risk among T2DM patients. We firstly recommend that statin treatment be considered in patients with T2DM to reduce their risk of developing atherosclerotic CVDs. While evidence gathered in this study supports the use of statin therapy in T2DM, we recommend that future trials with large powered sample sizes and proper methodology be conducted over extended periods in adults with T2DM. These trials should also focus on standardizing the dosage and the form of statin that is more effective when giving a clear recommendation for T2DM. Considering CIMT as a surrogate marker when conducting future clinical trials may assist in the development of new therapies against CVD and associated complications.

## Figures and Tables

**Table 1:** General characteristics of included studies.

Author	Country	Study Design	Population	Interventions	Baseline Age (Years)	Male (%) Female (%)	BMI in Statin Group (kg·m^−2^)	Main Results
Beishuizen et al., 2004 [40]	The Netherlands	Randomized, double-blinded control trial	103 T2DM patients on Simvastatin	Simvastatin at 20 mg for 24 months	58.8 ± 11.3	61 (59) 42 (41)	31.0 ± 6.0	No significant difference in carotid intima–media thickness (CIMT) between baseline and post-treatment.
Takahashi et al., 2004a [46]	Japan	Randomized controlled trial	15 T2DM patients on Simvastatin	Simvastatin at a dose of 5–10 mg for 12 months	59.1 ± 5.5	NR	22.8 ± 2.7	CIMT increased following statin treatment.
Takahashi et al., 2004b [46]	Japan	Randomized controlled trial	15 T2DM patients on Pravastatin	Pravastatin at 10–20 mg for 12 months	57.9 ± 4.5	NR	24.4 ± 1.3	CIMT increased following statin treatment.
Akalin et al., 2008 [20]	Turkey	Prospective observational study	30 patients on Atorvastatin	Atorvastatin at 10 mg for 2.5 months	54.87 ± 8.0	15 (50) 15 (50)	28.92 ± 4.38	No significant difference in CIMT and CRP following statin treatment.
Fleg et al., 2008 [39]	United States of America	Randomized, double-blinded control trial	154 T2DM patients on unspecified statins	Statins for 36 months	56.9 ± 0.57	48 (31) 106 (69)	34 ± 0.36	C-reactive protein (CRP) and the CIMT were reduced in the treatment group.
Russell et al., 2010 [38]	United States of America	Randomized, double-blinded control trial	252 T2DM patients on Atorvastatin	Atorvastatin at 10 mg for 36 months	55.0 ± 9.0	85 (34) 167 (66)	33.5 ± 6.6	A reduction in CIMT and CRP was noted in participants on statin therapy compared to those in the control group.
Kadoglou et al., 2012a [43]	Greece	Randomized control trial	29 T2DM patients on Atorvastatin	Atorvastatin at 10 mg for 12 months	64.0 ± 5.9	18 (62) 11 (38)	29.76 ± 4.87	There was no significant change in CIMT after the treatment compared to baseline. However, CRP levels decreased after the treatment.
Kadoglou et al., 2012b [43]	Greece	Randomized control trial	19 T2DM patients on Atorvastatin	Atorvastatin at 80 mg for 12 months	65.2 ± 8.2	18 (95) 1 (5)	29.76 ± 4.87	CIMT and CRP levels were significantly reduced after 12 months of treatment.
Hong-Wei et al., 2015a [45]	China	Randomized controlled trial	45 T2DM patients on Atorvastatin	Atorvastatin at 20 mg for 24 weeks	57.0 ± 8.19	23 (51) 22(49)	25.42 ± 1.77	CIMT significantly decreased following statin treatment.
Hong-Wei et al., 2015b [45]	China	Randomized controlled trial	45 T2DM patients on Simvastatin	Simvastatin at 20 mg for 24 weeks	57.69 ± 8.34	20 (44) 25 (56)	25.69 ± 1.36	CIMT significantly decreased following statin treatment
Wang et al., 2017 [44]	China	Randomized controlled trial	49 T2DM patients on Atorvastatin	Atorvastatin at 20 mg for 12 months	58.0 ± 9.0	30 (61) 19 (39)	NR	CIMT and CRP significantly decreased after statin treatment.
Chen et al., 2018 [37]	China	Randomized, double-blinded control trial	46 T2DM patients on Atorvastatin	Atorvastatin at 20 mg for six months	82.9 ± 2.9	19 (41) 27 (59)	24.46 ± 2.61	CIMT and CRP were significantly reduced after six months of treatment.
Naresh et al., 2021 [41]	India	Randomized controlled trial	40 T2DM patients on Rosuvastatin	Rosuvastatin at 20 mg for 12 weeks	51.7 ± 6.8	40 (100) 0 (0)	27.32 ± 3.75	CIMT significantly decreased after statin treatment.
Ishigakhi et al., 2014a [42]	Japan	Prospective, randomized, open-label, parallel-group study	51 T2DM patients on Pitavastatin	Pitavastatin at 2 mg for 36 months	59.0 ± 8.8	21 (41) 30 (59)	25.4 ± 4.5	There was a significant reduction in CIMT levels in the statin group.
Ishigakhi et al., 2014b [42]	Japan	Prospective, randomized, open-label, parallel-group study	46 T2DM patients on Pravastatin	Pravastatin at 10 mg for 36 months	60.0 ± 9.6	24 (52) 22 (48)	26.0 ± 3.7	No significant difference in CIMT was observed post-treatment compared to baseline.

T2DM—type 2 diabetes mellitus; CRP—C-reactive protein; CIMT—carotid intima–media thickness; NR—not reported.

## Data Availability

All files supporting this are presented in Supplementary Information files.

## Supplementary Materials

The following supporting information can be downloaded at https://www.mdpi.com/article/10.3390/jcdd11090276/s1 Supplementary File S1: PRISMA checklist; Supplementary File S2: Table S1: Literature search on databases; Figure S1: Risk of bias assessment (ROB), A: Risk of bias traffic plot, B: Risk of bias summary plot; Figure S2: subgroup analysis of CIMT based on forms of statin treatments; Figure S3: Subgroup based on dosages of statin used on CIMT; Figure S4: Subgroup-analysis of CIMT based on continent of publication; Figure S5: Subgroup analysis of CIMT based on age of participants; Figure S6: Subgroup analysis based on form of statin on CRP; Figure S7: Subgroup analysis based on doses of statin on CRP; Figure S8: Subgroup analysis based on age of participants on CRP; Figure S9: Subgroup analysis based on continent of publication on CRP.
