# Supplementary material for: The Effect of Statins on Carotid Intima–Media Thickness and C–Reactive Protein in Type 2 Diabetes Mellitus: A Meta–Analysis
Source: J Cardiovasc Dev Dis. 2024 Sep 4;11(9):276. doi: 10.3390/jcdd11090276 (PMC11432595; doi:10.3390/jcdd11090276)
Supplement: Supplementary file 1 [file jcdd-11-00276-s001.zip › jcdd-3149835-supplementary File S2.pdf]

The Effect of Statins on Carotid Intima–Media Thickness and C–Reactive Protein in Type 2 Diabetes Mellitus: A Meta–Analysis

Given Reneilwe Mashaba [1,2,*], Wendy Nokhwezi Phoswa [1] and Kabelo Mokgalaboni [1,*]

[1] Department of Life and Consumer Science, College of Agriculture and Environmental Sciences, University of South Africa, Florida Campus, Roodepoort 1710, South Africa; phoswwn@unisa.ac.za
[2] DIMAMO Population Health Research Centre, University of Limpopo, Sovenga, Private Bag X1106, Polokwane 0727, South Africa
* Correspondence: given.mashaba@ul.ac.za (G.R.M.); mokgak@unisa.ac.za (K.M.)

Table S1: Literature search on databases.

**Database**	**Exact search**	**Number of records retrieved**
Scopus	(TITLE-ABS-KEY (statin) OR TITLE-ABS-KEY (atorvastatin) OR TITLE-ABS-KEY (pitavastatin) OR TITLE-ABS-KEY (pravastatin) OR TITLE-ABS-KEY (rosuvastatin) OR TITLE-ABS-KEY (cimvastatin) OR TITLE-ABS-KEY (lovastatin) OR TITLE-ABS-KEY (fluvastatin) OR TITLE-ABS-KEY (cerivastatin) AND TITLE-ABS-KEY (type 2 diabetes AND mellitus) AND TITLE-ABS-KEY (carotid AND intima AND media AND thickness) OR TITLE-ABS-KEY (cimt) AND TITLE-ABS-KEY (c-reactive AND protein) OR TITLE-ABS-KEY (crp)) AND (LIMIT-TO (DOCTYPE , "ar"))	25
PubMed	((((((((((((Statin[MeSH Terms]) OR (Atorvastatin[MeSH Terms])) OR (Pitavastatin[MeSH Terms])) OR (pravastatin[MeSH Terms])) OR (rosuvastatin[MeSH Terms])) OR (simvastatin[MeSH Terms])) OR (cimvastatin[MeSH Terms])) OR (fluvastatin[MeSH Terms])) OR (lovastatin[MeSH Terms])) AND (type 2 diabetes mellitus[MeSH Terms])) AND (carotid intima media thickness[MeSH Terms])) OR (CIMT[MeSH Terms])) AND (C-reactive protein[MeSH Terms])) OR (CRP[MeSH Terms])	1

PubMed	((statins) AND (carotid intima media thickness)) AND (type 2 diabetes mellitus)	44
PubMed	((Statins[MeSH Terms]) AND (C-reactive protein[MeSH Terms])) AND (type 2 diabetes mellitus[MeSH Terms])	69
Web of Science	((((((((((((((ALL=(statins)) OR ALL=(atorvastatine)) OR ALL=(pitavastatin)) OR ALL=(pravastatin)) OR ALL=(rosuvastatin)) OR ALL=(simvastatin)) OR ALL=(lovastatin)) OR ALL=(fluvastatin)) OR ALL=(fluvastatin)) OR ALL=(cerivastatin)) AND ALL=(type 2 diabetes mellitus)) OR ALL=(T2DM)) AND ALL=(carotid intima media-thickness)) OR ALL=(CIMT)) AND ALL=(C-reactive protein)) OR ALL=(CRP)) AND ALL=(Randomized case control)	353
Cochrane library	(type 2 diabetes mellitus):ti,ab,kw AND (statins):ti,ab,kw AND ("carotid intima media thickness"):ti,ab,kw AND ("C reactive protein"):ti,ab,kw	3

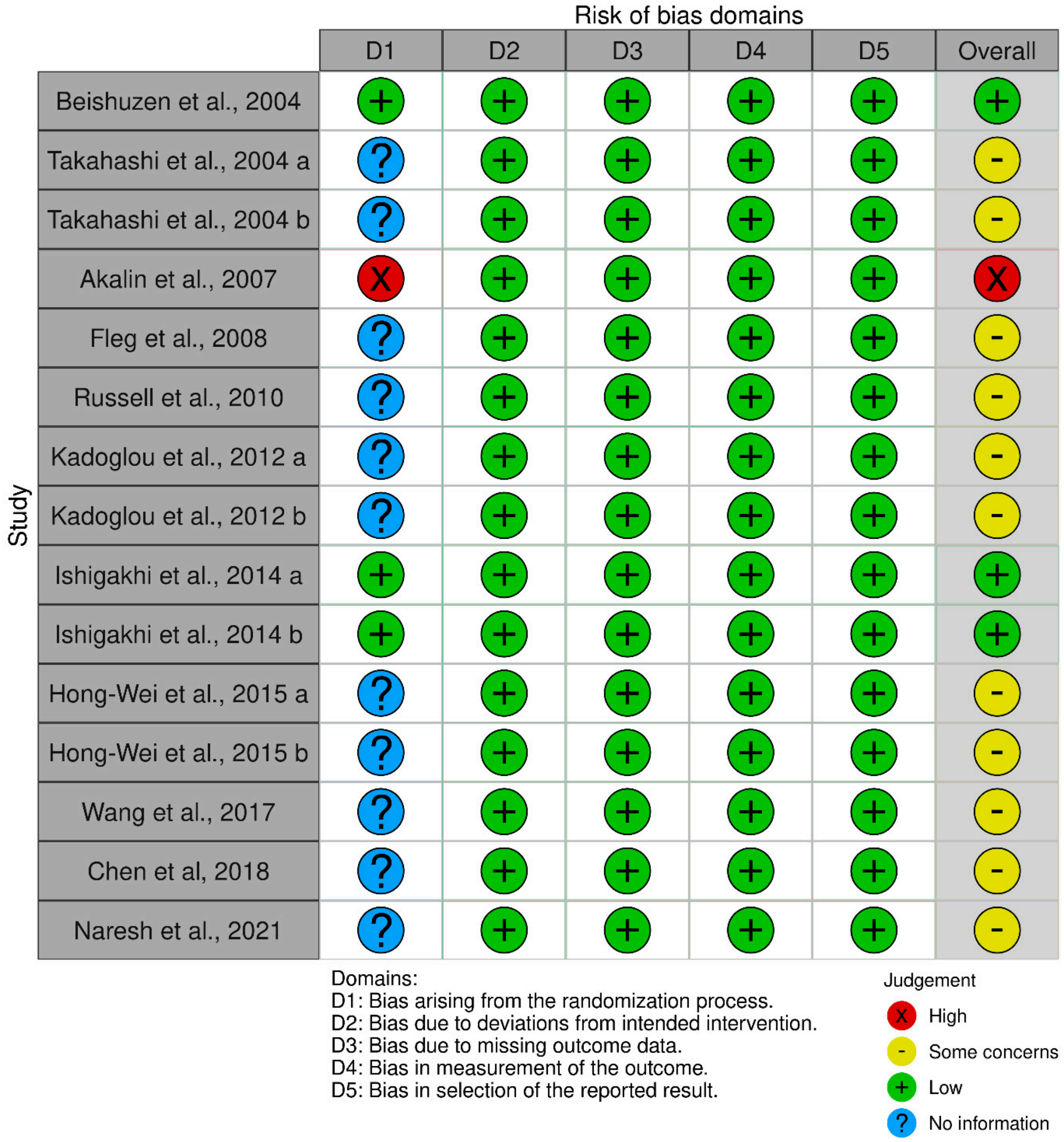
Risk of bias domains
D1
D2
D3
D4
D5
Overall
Study
Beishuzen et al., 2004
Takahashi et al., 2004 a
Takahashi et al., 2004 b
Akalin et al., 2007
Fleg et al., 2008
Russell et al., 2010
Kadoglou et al., 2012 a
Kadoglou et al., 2012 b
Ishigakhi et al., 2014 a
Ishigakhi et al., 2014 b
Hong-Wei et al., 2015 a
Hong-Wei et al., 2015 b
Wang et al., 2017
Chen et al, 2018
Naresh et al., 2021
Domains:
D1: Bias arising from the randomization process.
D2: Bias due to deviations from intended intervention.
D3: Bias due to missing outcome data.
D4: Bias in measurement of the outcome.
D5: Bias in selection of the reported result.
Judgement
High
Some concerns
Low
No information

A

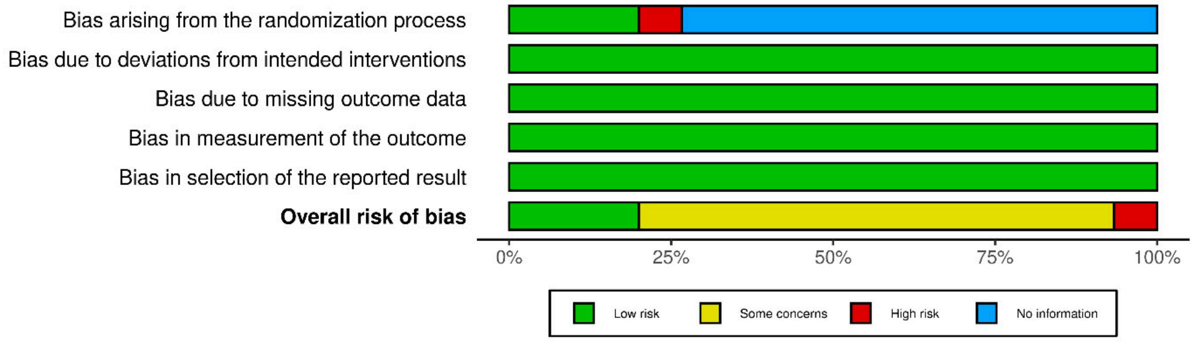
Bias arising from the randomization process
Bias due to deviations from intended interventions
Bias due to missing outcome data
Bias in measurement of the outcome
Bias in selection of the reported result
Overall risk of bias
0%
25%
50%
75%
100%
Low risk
Some concerns
High risk
No information

B

Figure S1: Risk of bias assessment (ROB), A: Risk of bias traffic plot, B: Risk of bias summary plot [20,37–46].

Study or Subgroup	Post-treatment Mean	Post-treatment SD	Post-treatment Total	Baseline Mean	Baseline SD	Baseline Total	Weight	Std. Mean Difference IV, Random, 95% CI
**1.1.1 Atorvastatin**								
Chen et al., 2018	1.25	0.11	46	1.31	0.12	46	7.4%	-0.52 [-0.93, -0.10]
Hong-Wei et al., 2015 a	0.17	0.06	45	0.91	0.77	45	7.1%	-1.34 [-1.80, -0.88]
Kadoglou et al., 2012 a	0.96	0.19	29	0.96	0.22	29	6.8%	0.00 [-0.51, 0.51]
Kadoglou et al., 2012 b	0.93	0.21	19	0.97	0.18	19	6.1%	-0.20 [-0.84, 0.44]
Russell et al., 2010	0.798	0.19	252	0.81	0.19	252	8.4%	-0.06 [-0.24, 0.11]
Wang et al., 2017	1.13	0.27	49	1.27	0.44	49	7.4%	-0.38 [-0.78, 0.02]
**Subtotal (95% CI)**			**440**			**440**	**43.3%**	**-0.41 [-0.80, -0.03]**
Heterogeneity: Tau² = 0.18; Chi² = 29.08, df = 5 (P < 0.0001); I² = 83%								
Test for overall effect: Z = 2.10 (P = 0.04)								
**1.1.2 Simvastatin**								
Beishuzen et al., 2004	0.765	0.116	103	0.763	0.124	103	8.0%	0.02 [-0.26, 0.29]
Hong-Wei et al., 2015 b	0.14	0.07	45	1.07	0.84	45	7.1%	-1.55 [-2.02, -1.07]
Takahashi et al., 2004 a	1.041	0.136	15	1.028	0.136	15	5.7%	0.09 [-0.62, 0.81]
**Subtotal (95% CI)**			**163**			**163**	**20.8%**	**-0.49 [-1.57, 0.59]**
Heterogeneity: Tau² = 0.84; Chi² = 32.91, df = 2 (P < 0.00001); I² = 94%								
Test for overall effect: Z = 0.88 (P = 0.38)								
**1.1.3 Rosuvastatin**								
Naresh et al., 2021	0.69	0.078	40	0.76	0.068	40	7.1%	-0.95 [-1.41, -0.48]
**Subtotal (95% CI)**			**40**			**40**	**7.1%**	**-0.95 [-1.41, -0.48]**
Heterogeneity: Not applicable								
Test for overall effect: Z = 4.01 (P < 0.0001)								
**1.1.4 Pravastatin**								
Ishigaki et al., 2012 b	0.834	0.247	46	0.905	0.304	46	7.4%	-0.25 [-0.66, 0.16]
Takahashi et al., 2004 b	1.099	0.153	15	1.079	0.153	15	5.7%	0.13 [-0.59, 0.84]
**Subtotal (95% CI)**			**61**			**61**	**13.1%**	**-0.16 [-0.52, 0.20]**
Heterogeneity: Tau² = 0.00; Chi² = 0.82, df = 1 (P = 0.37); I² = 0%								
Test for overall effect: Z = 0.88 (P = 0.38)								
**1.1.5 Pitavastatin**								
Ishigaki et al., 2012 a	0.802	0.236	51	0.861	0.249	51	7.5%	-0.24 [-0.63, 0.15]
**Subtotal (95% CI)**			**51**			**51**	**7.5%**	**-0.24 [-0.63, 0.15]**
Heterogeneity: Not applicable								
Test for overall effect: Z = 1.21 (P = 0.22)								
**1.1.6 Statin (unspecified)**								
Fleg et al., 2008	0.8009	0.0108	154	0.813	0.0108	154	8.2%	-1.12 [-1.36, -0.88]
**Subtotal (95% CI)**			**154**			**154**	**8.2%**	**-1.12 [-1.36, -0.88]**
Heterogeneity: Not applicable								
Test for overall effect: Z = 9.11 (P < 0.00001)								
**Total (95% CI)**			**909**			**909**	**100.0%**	**-0.47 [-0.77, -0.18]**
Heterogeneity: Tau² = 0.26; Chi² = 109.39, df = 13 (P < 0.00001); I² = 88%								
Test for overall effect: Z = 3.13 (P = 0.002)								
Test for subgroup differences: Chi² = 29.03, df = 5 (P < 0.0001), I² = 82.8%								

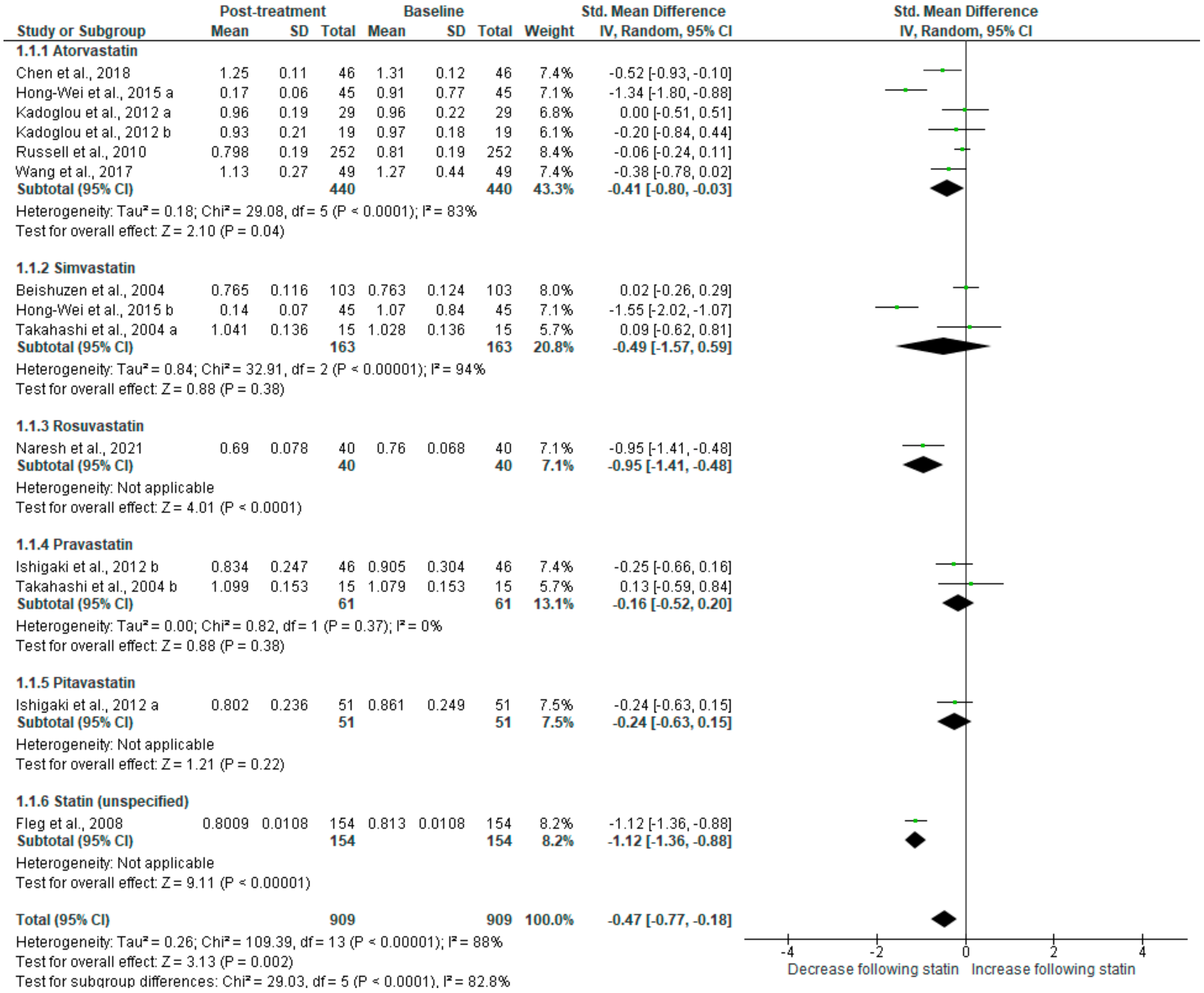

Figure S2: subgroup analysis of CIMT based on forms of statin treatments [20,37–46].

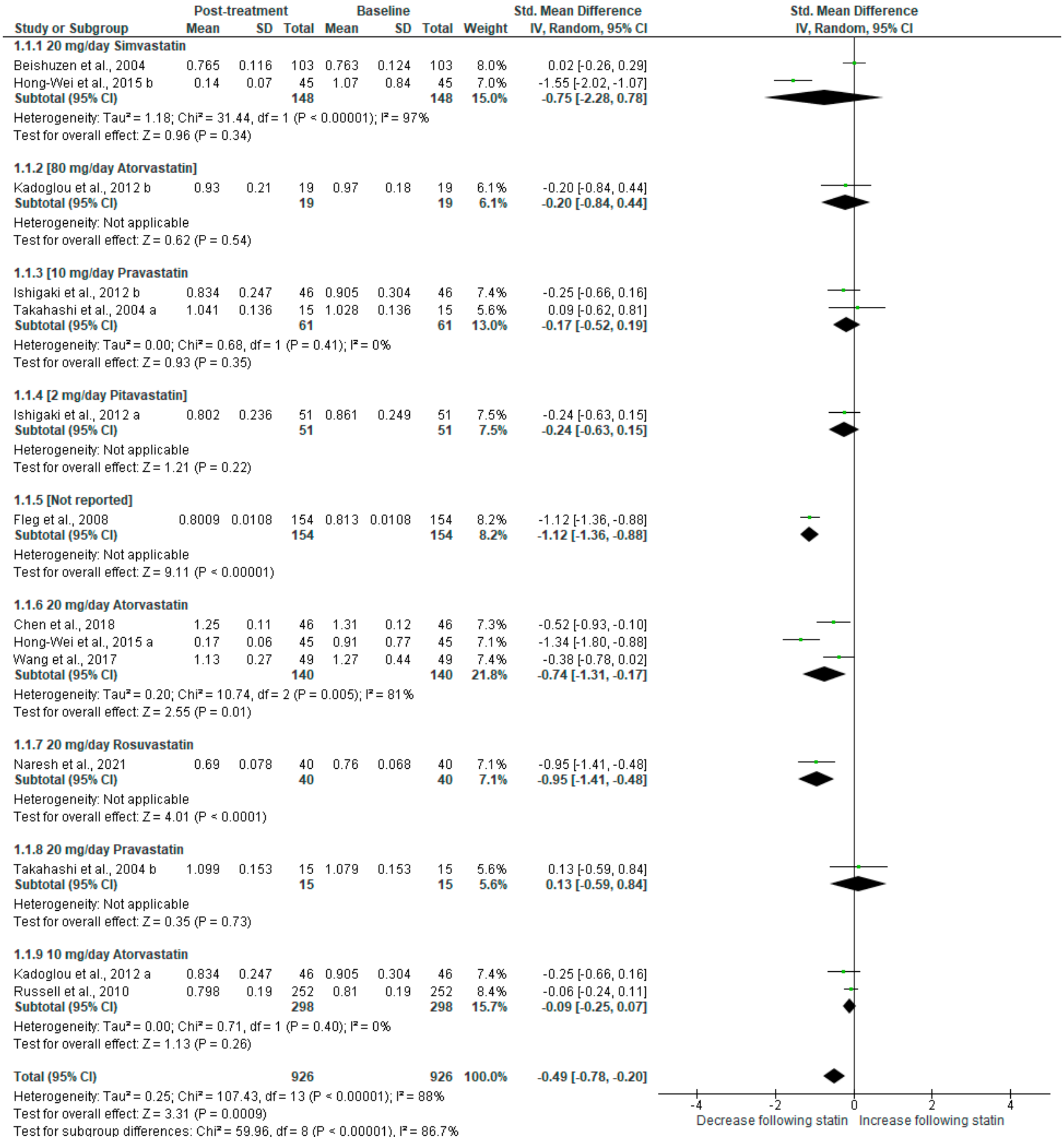

Figure S3: Subgroup based on dosages of statin used on CIMT [20,37–46].

Study or Subgroup	Post-treatment Mean	Post-treatment SD	Post-treatment Total	Baseline Mean	Baseline SD	Baseline Total	Weight	Std. Mean Difference IV, Random, 95% CI
**1.1.1 Asia**								
Chen et al., 2018	1.25	0.11	46	1.31	0.12	46	7.4%	-0.52 [-0.93, -0.10]
Hong-Wei et al., 2015 a	0.17	0.06	45	0.91	0.77	45	7.2%	-1.34 [-1.80, -0.88]
Hong-Wei et al., 2015 b	0.14	0.07	45	1.07	0.84	45	7.1%	-1.55 [-2.02, -1.07]
Ishigaki et al., 2012 a	0.834	0.247	46	0.905	0.304	46	7.5%	-0.25 [-0.66, 0.16]
Ishigaki et al., 2012 b	0.834	0.247	46	0.905	0.304	46	7.5%	-0.25 [-0.66, 0.16]
Naresh et al., 2021	0.69	0.078	40	0.76	0.068	40	7.2%	-0.95 [-1.41, -0.48]
Takahashi et al., 2004 a	1.041	0.136	15	1.028	0.136	15	5.8%	0.09 [-0.62, 0.81]
Takahashi et al., 2004 b	1.099	0.153	15	1.079	0.153	15	5.8%	0.13 [-0.59, 0.84]
Wang et al., 2017	1.13	0.27	49	1.27	0.44	49	7.5%	-0.38 [-0.78, 0.02]
**Subtotal (95% CI)**			**347**			**347**	**62.8%**	**-0.59 [-0.95, -0.22]**
Heterogeneity: Tau² = 0.24; Chi² = 41.87, df = 8 (P < 0.00001); I² = 81%								
Test for overall effect: Z = 3.17 (P = 0.002)								
**1.1.2 Europe**								
Beishuzen et al., 2004	0.765	0.116	103	0.763	0.124	103	8.1%	0.02 [-0.26, 0.29]
Kadoglou et al., 2012 a	0.93	0.21	19	0.97	0.18	19	6.2%	-0.20 [-0.84, 0.44]
Kadoglou et al., 2012 b	0.93	0.21	19	0.97	0.18	19	6.2%	-0.20 [-0.84, 0.44]
**Subtotal (95% CI)**			**141**			**141**	**20.5%**	**-0.04 [-0.28, 0.19]**
Heterogeneity: Tau² = 0.00; Chi² = 0.65, df = 2 (P = 0.72); I² = 0%								
Test for overall effect: Z = 0.35 (P = 0.73)								
**1.1.3 America**								
Fleg et al., 2008	0.8009	0.0108	154	0.813	0.0108	154	8.2%	-1.12 [-1.36, -0.88]
Russell et al., 2010	0.798	0.19	252	0.81	0.19	252	8.4%	-0.06 [-0.24, 0.11]
**Subtotal (95% CI)**			**406**			**406**	**16.7%**	**-0.59 [-1.62, 0.45]**
Heterogeneity: Tau² = 0.54; Chi² = 48.39, df = 1 (P < 0.00001); I² = 98%								
Test for overall effect: Z = 1.11 (P = 0.27)								
**Total (95% CI)**			**894**			**894**	**100.0%**	**-0.49 [-0.78, -0.19]**
Heterogeneity: Tau² = 0.26; Chi² = 107.02, df = 13 (P < 0.00001); I² = 88%								
Test for overall effect: Z = 3.24 (P = 0.001)								
Test for subgroup differences: Chi² = 6.64, df = 2 (P = 0.04), I² = 69.9%								

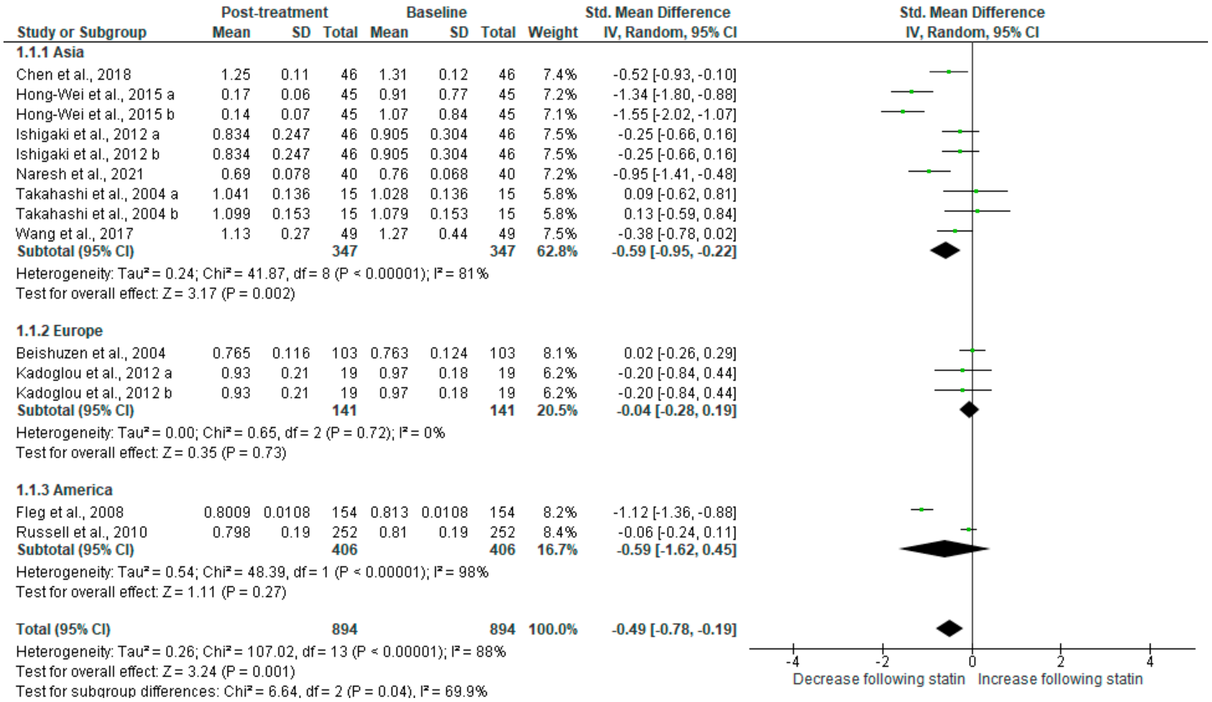

Figure S4: Subgroup-analysis of CIMT based on continent of publication [20,37–46].

Study or Subgroup	Post-treatment Mean	Post-treatment SD	Post-treatment Total	Baseline Mean	Baseline SD	Baseline Total	Weight	Std. Mean Difference IV, Random, 95% CI
**1.1.1 Below 60 years old**								
Beishuzen et al., 2004	0.765	0.116	103	0.763	0.124	103	8.1%	0.02 [-0.26, 0.29]
Fleg et al., 2008	0.8009	0.0108	154	0.813	0.0108	154	8.2%	-1.12 [-1.36, -0.88]
Hong-Wei et al., 2015 a	0.17	0.06	45	0.91	0.77	45	7.2%	-1.34 [-1.80, -0.88]
Hong-Wei et al., 2015 b	0.14	0.07	45	1.07	0.84	45	7.1%	-1.55 [-2.02, -1.07]
Ishigaki et al., 2012 a	0.834	0.247	46	0.905	0.304	46	7.5%	-0.25 [-0.66, 0.16]
Naresh et al., 2021	0.69	0.078	40	0.76	0.068	40	7.2%	-0.95 [-1.41, -0.48]
Russell et al., 2010	0.798	0.19	252	0.81	0.19	252	8.4%	-0.06 [-0.24, 0.11]
Takahashi et al., 2004 a	1.041	0.136	15	1.028	0.136	15	5.8%	0.09 [-0.62, 0.81]
Takahashi et al., 2004 b	1.099	0.153	15	1.079	0.153	15	5.8%	0.13 [-0.59, 0.84]
Wang et al., 2017	1.13	0.27	49	1.27	0.44	49	7.5%	-0.38 [-0.78, 0.02]
**Subtotal (95% CI)**			**764**			**764**	**72.7%**	**-0.56 [-0.94, -0.17]**
Heterogeneity: Tau² = 0.33; Chi² = 104.97, df = 9 (P < 0.00001); I² = 91%								
Test for overall effect: Z = 2.83 (P = 0.005)								
**1.1.2 Above 60 years old**								
Chen et al., 2018	1.25	0.11	46	1.31	0.12	46	7.4%	-0.52 [-0.93, -0.10]
Ishigaki et al., 2012 b	0.834	0.247	46	0.905	0.304	46	7.5%	-0.25 [-0.66, 0.16]
Kadoglou et al., 2012 a	0.93	0.21	19	0.97	0.18	19	6.2%	-0.20 [-0.84, 0.44]
Kadoglou et al., 2012 b	0.93	0.21	19	0.97	0.18	19	6.2%	-0.20 [-0.84, 0.44]
**Subtotal (95% CI)**			**130**			**130**	**27.3%**	**-0.33 [-0.57, -0.08]**
Heterogeneity: Tau² = 0.00; Chi² = 1.23, df = 3 (P = 0.75); I² = 0%								
Test for overall effect: Z = 2.64 (P = 0.008)								
**Total (95% CI)**			**894**			**894**	**100.0%**	**-0.49 [-0.78, -0.19]**
Heterogeneity: Tau² = 0.26; Chi² = 107.02, df = 13 (P < 0.00001); I² = 88%								
Test for overall effect: Z = 3.24 (P = 0.001)								
Test for subgroup differences: Chi² = 0.94, df = 1 (P = 0.33), I² = 0%								

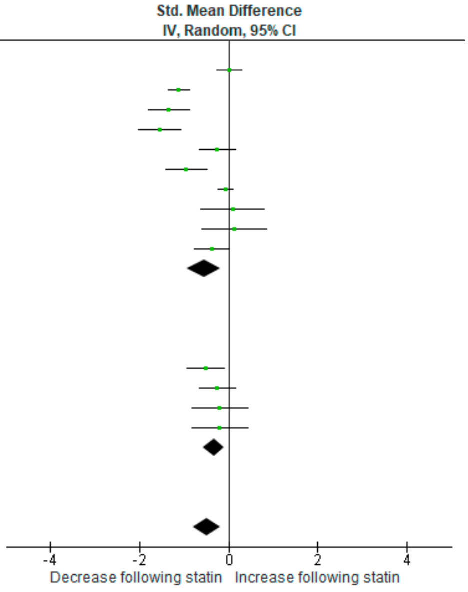

Figure S5: Subgroup analysis of CIMT based on age of participants [20,37–46].

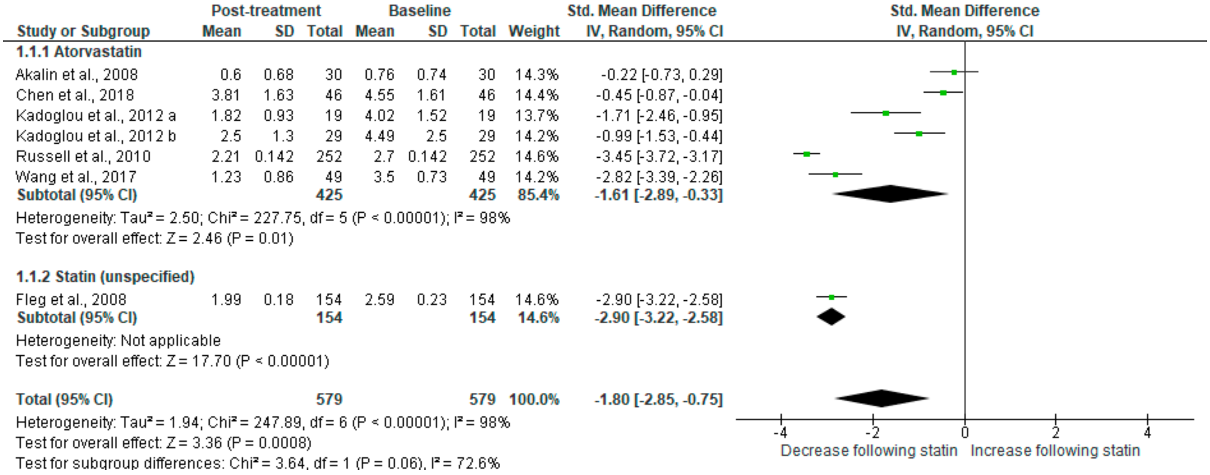

Figure S6: Subgroup analysis based on form of statin on CRP [20,37–39,43,44].

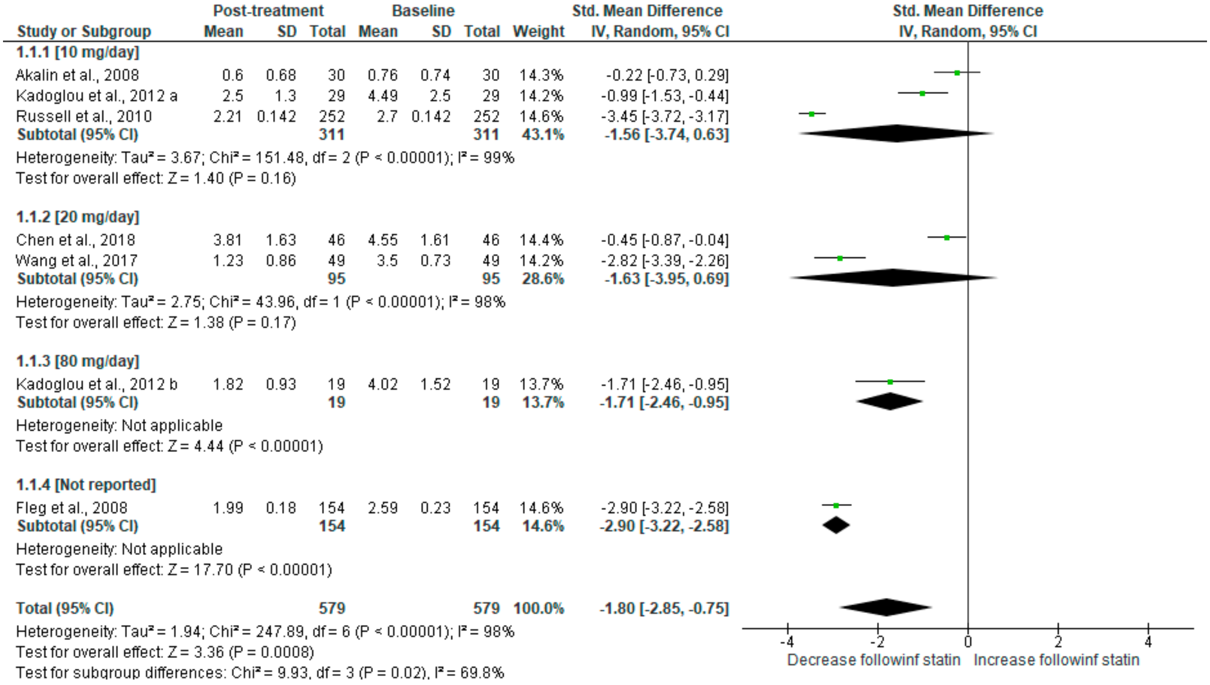

Figure S7: Subgroup analysis based on doses of statin on CRP [20,37–39,43,44].

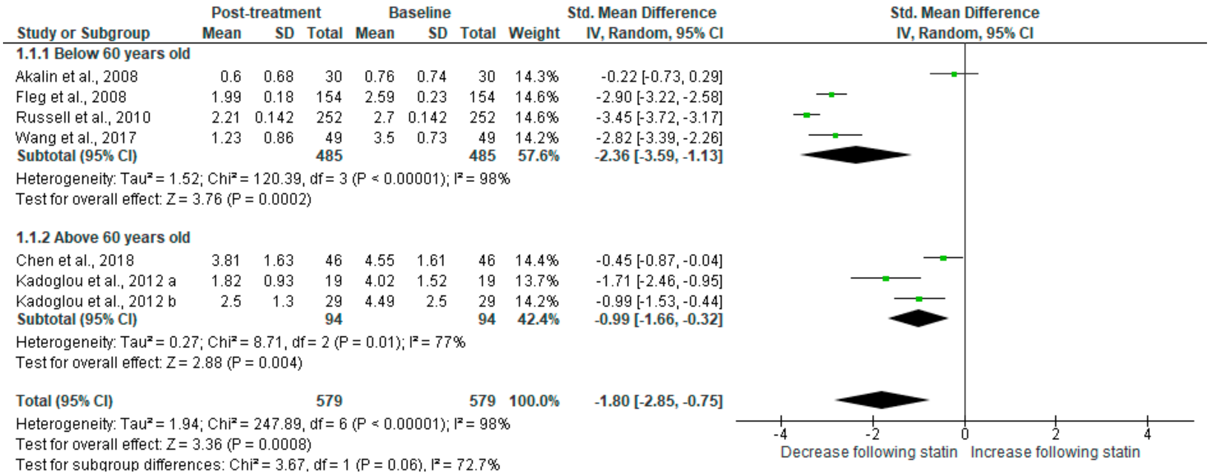

Study or Subgroup	Post-treatment Mean	SD	Total	Baseline Mean	SD	Total	Weight	Std. Mean Difference IV, Random, 95% CI
**1.1.1 Below 60 years old**								
Akalin et al., 2008	0.6	0.68	30	0.76	0.74	30	14.3%	-0.22 [-0.73, 0.29]
Fleg et al., 2008	1.99	0.18	154	2.59	0.23	154	14.6%	-2.90 [-3.22, -2.58]
Russell et al., 2010	2.21	0.142	252	2.7	0.142	252	14.6%	-3.45 [-3.72, -3.17]
Wang et al., 2017	1.23	0.86	49	3.5	0.73	49	14.2%	-2.82 [-3.39, -2.26]
**Subtotal (95% CI)**			**485**			**485**	**57.6%**	**-2.36 [-3.59, -1.13]**
Heterogeneity: Tau² = 1.52; Chi² = 120.39, df = 3 (P < 0.00001); I² = 98%								
Test for overall effect: Z = 3.76 (P = 0.0002)								
**1.1.2 Above 60 years old**								
Chen et al., 2018	3.81	1.63	46	4.55	1.61	46	14.4%	-0.45 [-0.87, -0.04]
Kadoglou et al., 2012 a	1.82	0.93	19	4.02	1.52	19	13.7%	-1.71 [-2.46, -0.95]
Kadoglou et al., 2012 b	2.5	1.3	29	4.49	2.5	29	14.2%	-0.99 [-1.53, -0.44]
**Subtotal (95% CI)**			**94**			**94**	**42.4%**	**-0.99 [-1.66, -0.32]**
Heterogeneity: Tau² = 0.27; Chi² = 8.71, df = 2 (P = 0.01); I² = 77%								
Test for overall effect: Z = 2.88 (P = 0.004)								
**Total (95% CI)**			**579**			**579**	**100.0%**	**-1.80 [-2.85, -0.75]**
Heterogeneity: Tau² = 1.94; Chi² = 247.89, df = 6 (P < 0.00001); I² = 98%								
Test for overall effect: Z = 3.36 (P = 0.0008)								
Test for subgroup differences: Chi² = 3.67, df = 1 (P = 0.06), I² = 72.7%								

Figure S8: Subgroup analysis based on age of participants on CRP [20,37–39,43,44].

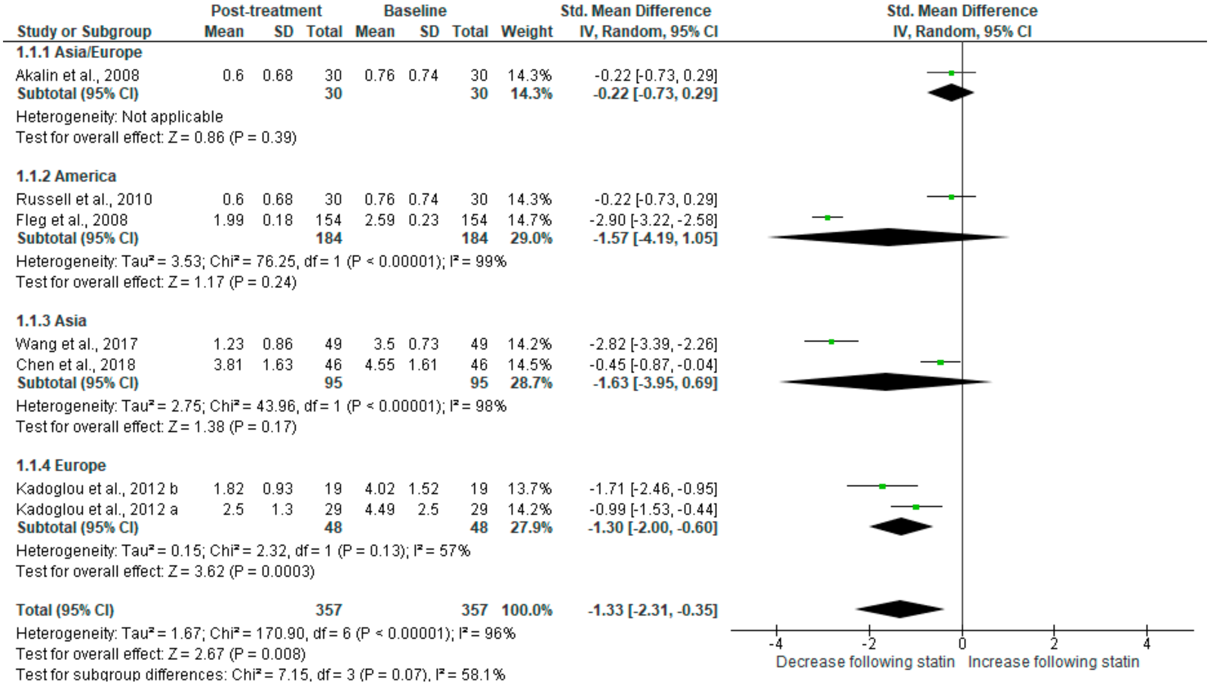

Study or Subgroup	Post-treatment Mean	SD	Total	Baseline Mean	SD	Total	Weight	Std. Mean Difference IV, Random, 95% CI
**1.1.1 Asia/Europe**								
Akalin et al., 2008	0.6	0.68	30	0.76	0.74	30	14.3%	-0.22 [-0.73, 0.29]
**Subtotal (95% CI)**			**30**			**30**	**14.3%**	**-0.22 [-0.73, 0.29]**
Heterogeneity: Not applicable								
Test for overall effect: Z = 0.86 (P = 0.39)								
**1.1.2 America**								
Russell et al., 2010	0.6	0.68	30	0.76	0.74	30	14.3%	-0.22 [-0.73, 0.29]
Fleg et al., 2008	1.99	0.18	154	2.59	0.23	154	14.7%	-2.90 [-3.22, -2.58]
**Subtotal (95% CI)**			**184**			**184**	**29.0%**	**-1.57 [-4.19, 1.05]**
Heterogeneity: Tau² = 3.53; Chi² = 76.25, df = 1 (P < 0.00001); I² = 99%								
Test for overall effect: Z = 1.17 (P = 0.24)								
**1.1.3 Asia**								
Wang et al., 2017	1.23	0.86	49	3.5	0.73	49	14.2%	-2.82 [-3.39, -2.26]
Chen et al., 2018	3.81	1.63	46	4.55	1.61	46	14.5%	-0.45 [-0.87, -0.04]
**Subtotal (95% CI)**			**95**			**95**	**28.7%**	**-1.63 [-3.95, 0.69]**
Heterogeneity: Tau² = 2.75; Chi² = 43.96, df = 1 (P < 0.00001); I² = 98%								
Test for overall effect: Z = 1.38 (P = 0.17)								
**1.1.4 Europe**								
Kadoglou et al., 2012 b	1.82	0.93	19	4.02	1.52	19	13.7%	-1.71 [-2.46, -0.95]
Kadoglou et al., 2012 a	2.5	1.3	29	4.49	2.5	29	14.2%	-0.99 [-1.53, -0.44]
**Subtotal (95% CI)**			**48**			**48**	**27.9%**	**-1.30 [-2.00, -0.60]**
Heterogeneity: Tau² = 0.15; Chi² = 2.32, df = 1 (P = 0.13); I² = 57%								
Test for overall effect: Z = 3.62 (P = 0.0003)								
**Total (95% CI)**			**357**			**357**	**100.0%**	**-1.33 [-2.31, -0.35]**
Heterogeneity: Tau² = 1.67; Chi² = 170.90, df = 6 (P < 0.00001); I² = 96%								
Test for overall effect: Z = 2.67 (P = 0.008)								
Test for subgroup differences: Chi² = 7.15, df = 3 (P = 0.07), I² = 58.1%								

Figure S9: Subgroup analysis based on continent of publication on CRP [20,37–39,43,44].

Reference

20. Akalin, A.; Temiz, G.; Akcar, N.; Sensoy, B. Short Term Effects of Atorvastatin on Endothelial Functions and Oxidized LDL Levels in Patients with Type 2 Diabetes. Endocr. J. 2008, 55, 861–866. https://doi.org/10.1507/endocrj.k07e-121.

37. Chen, W.; Tian, T.; Wang, S.; Xue, Y.; Sun, Z.; Wang, S. Characteristics of Carotid Atherosclerosis in Elderly Patients with Type 2 Diabetes at Different Disease Course, and the Intervention by Statins in Very Elderly Patients. J. Diabetes Investig. 2018, 9, 389–395. https://doi.org/10.1111/jdi.12710.

38. Russell, M.; Silverman, A.; Fleg, J.L.; Lee, E.T.; Mete, M.; Weir, M.; Wilson, C.; Yeh, F.; Howard, B.V.; Howard, W.J. Achieving Lipid Targets in Adults with Type 2 Diabetes: The Stop Atherosclerosis in Native Diabetics Study. J. Clin. Lipidol. 2010, 4, 435–443. https://doi.org/10.1016/j.jacl.2010.07.007.

39. Fleg, J.L.; Mete, M.; Howard, B.V.; Umans, J.G.; Roman, M.J.; Ratner, R.E.; Silverman, A.; Galloway, J.M.; Henderson, J.A.; Weir, M.R.; et al. Effect of Statins Alone Versus Statins Plus Ezetimibe on Carotid Atherosclerosis in Type 2 Diabetes. The SANDS (Stop Atherosclerosis in Native Diabetics Study) Trial. J. Am. Coll. Cardiol. 2008, 52, 2198–2205. https://doi.org/10.1016/j.jacc.2008.10.031.

40. Beishuizen, E.D.; Tamsma, J.T.; Jukema, J.W.; van de Ree, M.A.; van der Vijver, J.C.M.; Meinders, A.E.; Huisman, M. V The Effect of Statin Therapy on Endothelial Function in Type 2 Diabetes without Manifest Cardiovascular Disease. Diabetes Care 2005, 28, 1668–1674. https://doi.org/10.2337/diacare.28.7.1668.

41. Naresh, S.; Bitla, A.R.; Rao, P.V.L.N.S.; Sachan, A.; Amancharla, Y.L. Efficacy of Oral Rosuvastatin Intervention on HDL and Its Associated Proteins in Men with Type 2 Diabetes Mellitus. Endocrine 2021, 71, 76–86. https://doi.org/10.1007/s12020-020-02472-5.

42. Ishigaki, Y.; Kono, S.; Katagiri, H.; Oka, Y.; Oikawa, S.; Investigators, N. Elevation of HDL-C in Response to Statin Treatment Is Involved in the Regression of Carotid Atherosclerosis. J. Atheroscler. Thromb. 2014, 21, 1055–1065. https://doi.org/10.5551/jat.22095.

43. Kadoglou, N.P.E.; Sailer, N.; Kapelouzou, A.; Lampropoulos, S.; Vitta, I.; Kostakis, A.; Liapis, C.D. Effects of Atorvastatin on Apelin, Visfatin (Nampt), Ghrelin and Early Carotid Atherosclerosis in Patients with Type 2 Diabetes. Acta Diabetol. 2012, 49, 269–276. https://doi.org/10.1007/s00592-011-0310-0.

44. Wang, J.; Ai, X.-B.; Wang, F.; Zou, Y.-W.; Li, L.; Yi, X.-L. Efficacy of Ezetimibe Combined with Atorvastatin in the Treatment of Carotid Artery Plaque in Patients with Type 2 Diabetes Mellitus Complicated with Coronary Heart Disease. Int. Angiol. 2017, 36, 467–473. https://doi.org/10.23736/S0392-9590.17.03818-4.

45. Hong-Wei, M.; Wei-Dong, R.; Jun-Ming, W.; Hong-Qiang, L.; Li-Mei, H.; Qiu-Zi, Z. The Effects of Atorvastatin on the Level of Endothelial Microparticles and Carotid Intima Media Thickness in Type 2 Diabetes Mellitus. Arteriosclerosis 2015, 23, 829–833.

46. Takahashi, T.; Ishii, N.; Itai, K.; Goto, R.; Higashi, K.; Kobori, S. HMG-CoA Reductase Inhibitors Suppress the Development and Progres-Sion of Carotid Artery Intimal-Medial Thickening in Hypercholesterolemic Type 2 Diabetic Patients. J. Atheroscler. Thromb. 2004, 12, 149–153.
